# Sponge-like CoNi Catalysts Synthesized by Combustion of Reactive Solutions: Stability and Performance for CO_2_ Hydrogenation

**DOI:** 10.3390/ma15155129

**Published:** 2022-07-23

**Authors:** Nikolay Evdokimenko, Zhanna Yermekova, Sergey Roslyakov, Olga Tkachenko, Gennady Kapustin, Denis Bindiug, Alexander Kustov, Alexander S. Mukasyan

**Affiliations:** 1 Center of Functional Nano-Ceramics, National University of Science and Technology “MISiS”, 119049 Moscow, Russia; nikolayevdokimenko@bk.ru (N.E.); ifjanna@gmail.com (Z.Y.); denis-bindyug@yandex.ru (D.B.); kyst@list.ru (A.K.); 2 N.D. Zelinsky Institute of Organic Chemistry RAS, 119991 Moscow, Russia; ot113@mail.ru (O.T.); gik@server.ioc.ac.ru (G.K.); 3 Department of Chemistry, M. V. Lomonosov Moscow State University, 119991 Moscow, Russia; 4 Department of Chemical and Biomolecular Engineering, University of Notre Dame, Notre Dame, IN 46556, USA

**Keywords:** combustion, CoNi alloys, catalysis, CO_2_ hydrogenation, methanation

## Abstract

Active and stable catalysts are essential for effective hydrogenation of gaseous CO_2_ into valuable chemicals. This work focuses on the structural and catalytic features of single metals, i.e., Co and Ni, as well as bimetallic CoNi alloy catalysts synthesized via combustion of reactive sol-gels. Different characterization methods were used for studying the relationships between the structure, composition, and catalytic activity of the fabricated materials. All catalysts exhibited highly porous sponge-like microstructure. The outermost surfaces of the CoNi alloys were more saturated with Co, while a stoichiometric Co/Ni ratio was observed for the particle’s bulk. Catalytic properties of the as-synthesized powders were studied in the CO_2_ hydrogenation reaction at 300 °C for over 80 h of time on stream. All the catalysts demonstrated exceptional selectivity with respect to CH_4_ formation. However, the combination of elemental Co and Ni in a single phase resulted in a synergistic effect in bulk alloy catalysts, with activity twofold to threefold that of single-metal catalysts. The activity and stability of the CoNi_3_ catalyst were higher than those previously reported for Ni-based catalysts. The reasons for this behavior are discussed.

## 1. Introduction

Reducing CO_2_ emission into the atmosphere is a critical, climate-defining issue that has been a topic of significant interest during the last decade [1,2,3]. Conversion of CO_2_ into synthetic natural gas (syngas) through its catalytic hydrogenation (Equation (1)) is considered promising for the recycling of captured or newly produced CO_2_, although many other valuable chemicals could also be produced using this approach [4,5,6,7].

CO_2_ + 4H_2_ ⇄ CH_4_ + 2H_2_O, ΔH_298_K = −165.0 kJ/mol (1)

Noble and rare-earth metal-based catalysts are known for their high efficiency with respect to CO_2_ hydrogenation, but the growing demand for these catalysts has prompted the search for more commercially appealing materials [8,9]. Transition metals have attracted significant attention as cost-efficient alternatives [10]. Among these, Ni exhibits exceptional selectivity for the conversion of CO_2_ into CH_4_, but has some drawbacks, such as the relatively low lifetime of the catalysts owing to the sintering, oxidation, and carbonization of the metal active sites [11].

The specific interaction of metal particles with the supporting material is a viable approach for stabilizing active Ni sites against sintering and oxidation. The effect of the supporting material on the Ni catalyst modification has been intensely studied for single-phase substrates such as Al_2_O_3_ [12,13], TiO_2_ [14,15], SiO_2_ [16,17,18] CeO_2_ [12,19,20], ZrO_2_ [12,21], and hybrid materials with or without additional catalytically active dopants [22,23,24,25,26,27]. In all cases, however, there is a significant difference between the thermal conductivities of Ni (90.7 W/m·K) and ceramic supports (e.g., that of CeO_2_, at 8 W/m·K [28]), which often leads to the formation of local hot spots and subsequent carbonization of active sites [11,29].

The catalyst properties can be improved by controlling the catalyst composition, using, for example, bimetallic phases [30,31,32]. Specifically, Co has been a promising additive for modifying the electronic and crystal structures of Ni. For example, it was reported that the formation of a homogeneous Ni-Co alloy led to good CO conversion and CH_4_ selectivity at temperatures below 380 °C [29]. The electron enrichment of the Ni-Co alloy contributed to the CO_2_ adsorption, CO dissociation, and H_2_ spillover, which facilitated CH_4_ formation and the removal of deposited C species.

Recently, increasing attention has been paid to the development of structural catalysts, with the catalysts’ surface texture [33,34] and morphology as the key parameters for improving their performance and stability [35,36,37,38]. Sponge-like structured catalysts are highly advantageous, owing to their high surface-to-volume ratio and good mass and heat transfer at low-pressure drops [8,39,40]. The implementation of transition-metal foams in the CO_2_ hydrogenation reaction may significantly improve the heat transfer from the overheated spots through the thin walls of the sponge framework, thereby increasing the catalyst stability [41].

Sol-gel combustion synthesis (SGCS) is a unique approach for tailoring the morphology of a material, and allows the fabrication of metallic foams with controlled pore-size distribution [42,43,44,45]. In this work, we studied the synergetic effect of Co-Ni bonds in bulk Co-Ni alloys on the CO_2_ conversion reaction run on sponge-like structural metal catalysts fabricated by SGCS. Different Co_x_Ni_1−x_ compositions were synthesized, with x parameter values of 1, 0.75, 0.5, 0.25, and 0. The relationships between the structure, composition, and catalytic performance of the materials for CO_2_ hydrogenation were investigated at 300 °C for up to 80 h of time on stream (TOS).

## 2. Materials and Methods

### 2.1. Preparation of Catalysts

The catalysts were synthesized via the SGCS of a reactive mixture of metal nitrates and organic fuels [45]. Specifically, Co(NO_3_)_2_·6H_2_O and Ni(NO_3_)_2_·6H_2_O nitrates were used as metal-containing sources/oxidizers for fabricating single-metal (Ni or Co) and bimetallic (CoNi_3_, CoNi, and Co_3_Ni) materials. Aminoacetic acid (C_2_H_5_NO_2_) was used as a structure-forming reducer/fuel. All chemicals (Chimmed, Moscow, Russia) were 98% purity grade. The fuel-to-oxidizer molar ratio, φ, was kept equal to 1.5 for all mixtures. It is worth noting that this ratio was selected based on our previous work [46] and allowed both the self-propagating reaction mode and the formation of pure metallic phases. For each reactive mixture, the metal nitrates were dissolved in 80 mL of distilled water, following which the corresponding amount of fuel was added. The obtained aqueous solution was mixed thoroughly for 30 min using a magnetic stirrer, until a homogeneous mixture was formed. Each reactive solution was kept in a furnace at 80 °C for 24 h to form a gel-like medium. The SGCS performed in an inert atmosphere of Ar inside a stainless-steel chemical reactor [45]. The combustion reaction was triggered using a resistively heated tungsten wire. After the local preheating of the reactive gel, the initiated combustion front propagated along the reactive medium in the self-sustained mode. In the combustion wave, the gel was converted into a solid product, which was investigated using various material characterization techniques.

### 2.2. Structural Characterization

The phase compositions of the powders were studied using X-ray diffraction (XRD). The XRD analysis was performed at room temperature using a DIFREY-401 diffractometer, operated at 25 kV and 40 mA using a Cu-Kα radiation source with a Bragg–Brentano focusing geometry. A JSM 7600F (JEOL, Akishima, Japan) field emission scanning electron microscope with a spatial resolution of ~1 nm, equipped with an elemental microanalysis system (EDX, Oxford Instruments, Abingdon-on-Thames, UK), was used for studying the product morphology and elemental ratios in the samples. XPS analysis was performed on an Axis Ultra DLD (Kratos Analytical, Manchester, UK) instrument with an Al-*K*α radiation source operating at 1456.6 eV. The binding energy of C1s (285 eV) was used as the internal standard. All spectra were recorded in the 20–1460 eV range. C 1s, Ni 2p, Co 2p, O 1s, and N 1s were recorded with 0.1 eV step sizes. The elemental composition of the catalysts was acquired in the outermost layer (thickness, 5–10 nm), and the atomic ratio of the elements was calculated from the integral line intensity corrected by the Scofield photoionization cross-section.

### 2.3. Performance Evaluation

The as-prepared bulk catalysts were studied for CO_2_ hydrogenation at a pressure of 2 MPa and temperature of 300 °C. A mixture of as-synthesized catalytically active metal particles, with diameters in the 50–100 μm range, and a quartz buffer powder (size, 100–500 µm) was prepared. Specifically, 0.2 g of the metallic catalyst was mixed with 0.8 g of quartz powder. To preserve the sponge-like structure of the catalysts, the mixture was not subjected to any additional mechanical treatment. Thus, the prepared mixture was loaded into a continuous-flow stainless-steel fixed-bed reactor (inner diameter, 4 mm). Before the catalytic reaction, each sample was reduced under the following conditions: temperature, 300 °C; heating rate, 10 °C/min; H_2_ flow rate, 50 mL/min; and run time, 1 h. After the catalyst activation, the feed gas was switched to a mixture of CO_2_ and H_2_ with a volume ratio of 1:4 and gas flow rates in the 3–12 mL/min range. Thus, the total volumetric flow rate of the initial mixture was 15 mL/min, while the volumetric hourly space velocity (VHSV) was 4500 mL g^−1^h^−1^. Before the actual measurements, each sample was kept under the above conditions for 2 h for reaching thermodynamic equilibrium.

Analysis of the reaction products was performed using a KRISTALL 5000 chromatograph equipped with three heat capacity detectors, one flame ionization detector, and three columns: M NaX 80/100 mesh 2 m/2 mm, HayeSep R 80/100 mesh 1 m/2 mm, and HayeSep Q 80/100 mesh 1 m/2 m, and with a capillary column MXT^®^-Alumina BOND/MAPD 30 m/0.53 mm.

The CO_2_ gas conversion was calculated as:

(2) $$K_{CO_{2}}=100\%\frac{\left(G_{s}X_{CO_{2}}^{0}-G_{f}X_{CO_{2}}\right)}{G_{s}X_{CO_{2}}^{0}}$$

where $G_{s}$ is the gas mixture flow (L/h) at the reactor inlet, $G_{f}$ is the gas mixture flow (L/h) at the reactor outlet, $X_{CO_{2}}^{0}$ is the CO_2_ fraction before the catalytic reaction, and $X_{CO_{2}}$ is the CO_2_ fraction after the catalysis.

Productivity of the CO_2_ hydrogenation was calculated as follows:

(3) $$r=\frac{(n{\left(CO_{2}\right)}_{s}-n\left(CO_{2}{)}_{f}\right)}{m_{cat}}$$

where $n{\left(CO_{2}\right)}_{s}$ is the inlet molar flow rate of CO_2_ (mol/h), $n{\left(CO_{2}\right)}_{f}$ is the outlet molar flow rate of CO_2_ (mol/h), and $m_{cat}$ is the total mass (kg) of the loaded catalyst.

The product formation selectivity without considering water formation was calculated as follows:

(4) $$S_{i}=\frac{Y_{i}}{\sum Y_{i}}$$

where $Y_{i}$ is the portion of the *i*th reaction product in the final flow after the catalytic hydrogenation process, while $S_{\mathrm{CO}}+S_{\mathrm{HC}}=100\%$ , where $S_{\mathrm{CO}}$ is the CO selectivity and $S_{\mathrm{HC}}$ is the C_1_–C_4_ formation selectivity.

The CH_4_ fraction ( $f_{{\mathrm{CH}}_{4}}$ ) from the total amount of product was calculated as follows:

(5) $$f_{{\mathrm{CH}}_{4}}=S_{{\mathrm{CH}}_{4}}/S_{\mathrm{HC}}$$

### 2.4. In Situ Diffuse Reflectance Infrared Fourier Transform Spectroscopy (DRIFTS) Experiments

In situ DRIFTS experiments were performed using an FTIR spectrometer (NICOLET “Protege” 460) equipped with a homemade diffuse reflectance attachment [47]. IR spectra were recorded in the 6000–400 cm^−1^ range at a resolution of 4 cm^−1^. To regulate the signal-to-noise ratio, 500 spectra were processed. Prior to the measurements, all of the samples were subjected to thermo-vacuum treatment at 300 °C (heating rate, 5 °C/min) and P = 0.13 Pa, for 2 h. The CO and CO_2_ adsorption occurred at room temperature (27 °C) in the P = 2.1–3 kPa and P = 1.3–2.4 kPa ranges, respectively. The absorption band intensity was measured according to the Kubelka–Munk theory. CaF_2_ powder was used as the reference material. Registration and spectral processing were performed using the OMNIC software. The CO and CO_2_ absorption spectra are shown as the differences between the data before and after adsorption. The 2338 cm^−1^ frequency shift of the ν_3_ CO_2_ absorption band with respect to the value in the free state was used as a standard [48].

### 2.5. Temperature-Programmed Reduction Experiments

H_2_-TPR measurements were performed using a semi-automatic setup with a thermal-conductivity detector. The specimen (mass, 100–150 mg) was placed in a quartz U-shaped reactor with a type-K thermocouple placed at the center of the sample. The sample was preliminarily outgassed under an Ar (30 mL/min) flow at 300 °C for 30 min, at a 10 °/min heating rate. Then, the sample was cooled to room temperature, and the feed gas was changed to a 5% H_2_/Ar mixture. After the stabilization of the baseline, the sample was heated to 850 °C, at a 10 °C/min heating rate. The water byproduct was removed by the trap placed between the reactor and the detector and cooled to −100 °C by a mixture of liquid N and ethanol. The katharometer signal and temperature were recorded on a computer using an analog-to-digital converter and Ekochrome software package. The detector was calibrated using CuO reduction (Aldrich-Chemie GmbH, 99%, St. Louis, MO, USA). All of the results were normalized to 1 g of the sample.

## 3. Results

### 3.1. Structural Characterization

**Figure 1:**
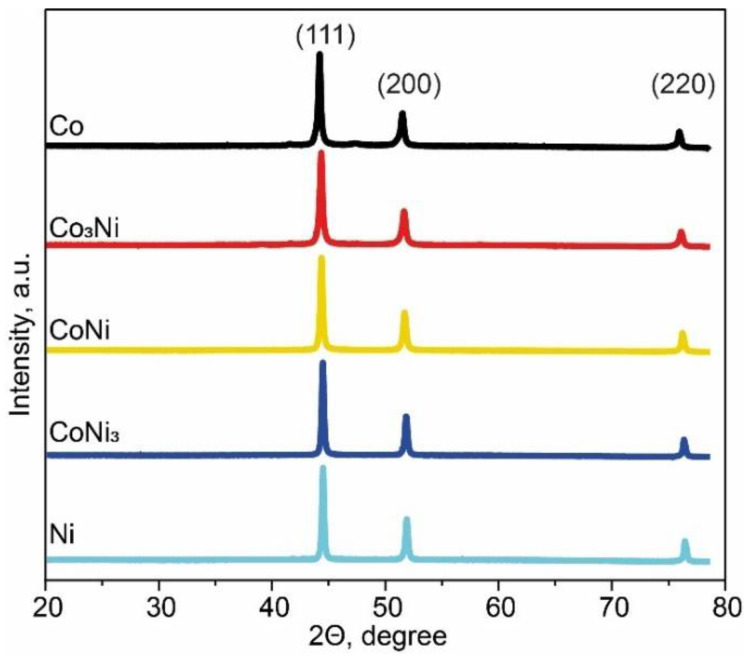
XRD patterns of the catalyst powders synthesized by SGCS.

The results of the XRD analysis of the SGCS catalysts are presented in Figure 1 and Table 1. According to the XRD data, the samples consisted of a single-metal phase (Co or Ni), or bimetallic Co_x_Ni_1−x_ phases (for x = 0.25, 0.5, 0.75), all with a face-centered-cubic (FCC) crystal structure (Fm-3m space group). The positions of the main peaks for the Co_3_Ni (44.349°), CoNi (44.365°), and CoNi_3_ (44.486°) samples were shifted to a range between the peaks of the constituent elements of Co (44.206°) and Ni (44.502°), which correspond to the JCPDS data for Co (PDF#15-0806) and Ni (PDF#04-0850). The observed changes in the peak positions of the bimetallic phases depending on the concentrations of Co and Ni agreed well with Vegard’s law and indicated the formation of disordered substitutional solid solutions (Co_x_Ni_1−x_) for a range of lattice parameters (Table 1) [49]. The XRD data indicated that no other crystalline phases, except for the desired stoichiometric composition, were formed. The crystallite size (D) for each material, calculated using the Scherrer equation, was in the 27–40 nm range, indicating a high degree of crystallinity.

**Figure 2:**
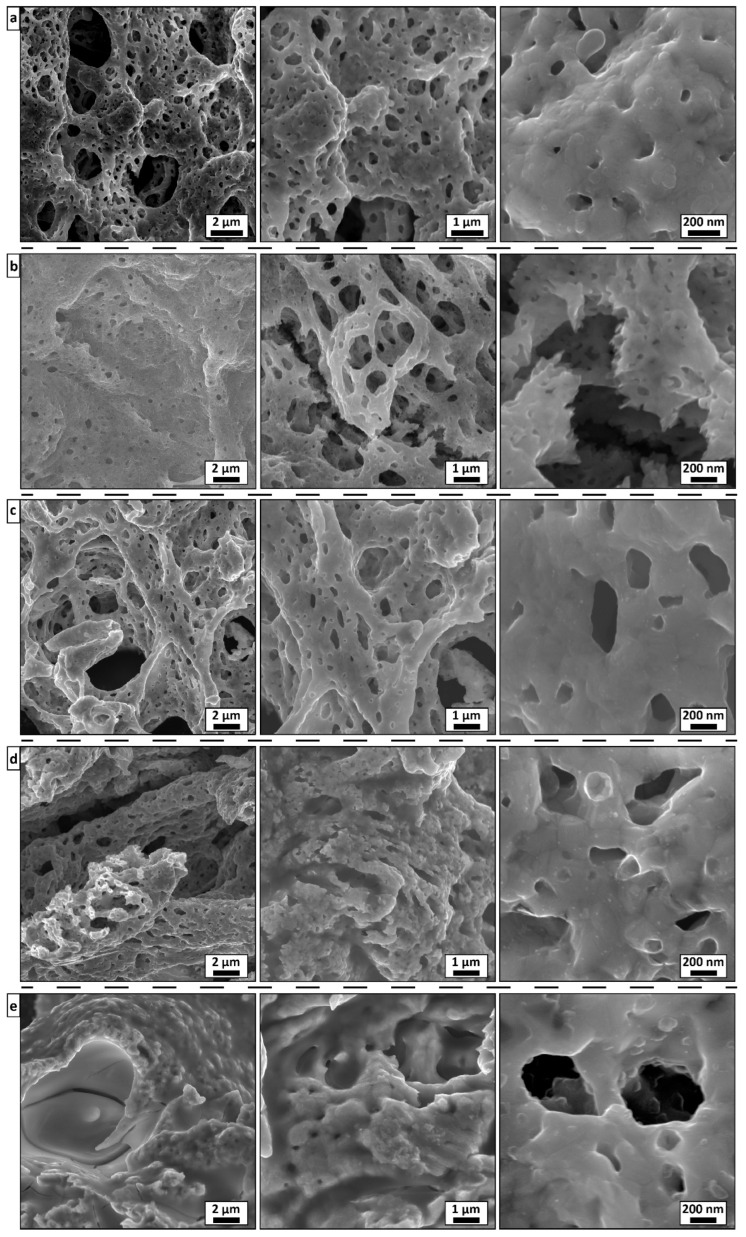
SEM images of (**a**) Co, (**b**) Co_3_Ni, (**c**) CoNi, (**d**) CoNi_3_, and (**e**) Ni bulk catalyst powders synthesized by SGCS.

The typical morphologies of the SGCS catalysts are shown in Figure 2. These materials are characterized by a highly porous microstructure, which generally resembles sponge-like agglomerations. The surfaces of the agglomerates depended on the catalyst composition and exhibited some unique patterns. Each material had cavities with a specific wall shape that had a visible number of open pores.

In the case of the Co catalyst (Figure 2a), the cavities pierced the body of the sample. Within the same particle, two types of pores were observed: larger pores (1–3 µm) and smaller submicrometer-scale pores (~300 nm). High-magnification images revealed that the walls featured many small pores with no strict underlying pattern, with the pores’ sizes in the 100–200 nm range. Following the addition of 25 at. % of Ni to Co (yielding the formation of the Co_3_Ni structure), the alloy’s microstructure completely lost the Co-specific surface pattern, with all of the cavities shrunk (Figure 2b). The alloy consisted of nanometerscale (~30 nm) crystals that agglomerated in large slices, with a relatively small number of pores. There was an empty space between the slices, where Co-like microstructures with large cavities elongated in one direction were present. For the equiatomic (CoNi) composition (Figure 2c), the microstructure of the particles resembled that of Co; however, it featured thicker walls and larger pores. When the amount of Co in the alloy was 25 at. % (Ni_3_Co), the microstructure of the agglomerates changed again, from a mono-carcass sponge to the one consisting of bridges, each of which with its own cavities and pores (Figure 2d). Moreover, small inclusions of nanoparticles could be distinguished over the entire bridge surface. At this point, the structure resembled the Ni microstructure consisting of thick bridges, chaotically interconnected with each other (Figure 2e). A closer examination revealed that the wall thickness of the Ni sponge was on the scale of a few micrometers, the surface was smooth in the middle and had many small outgrowth chips at the edges.

It is worth noting that in the case of supported catalysts, the structural porosity and specific surface area are primarily defined by the supporting material and do not depend much on the Co/Ni ratio [30,50,51,52]. In the case of SGCS-alloy catalysts, the structure is defined by catalytically active constituents, where the addition of Co to the Ni base leads to a visible increase in the porosity of the bulk alloys.

XPS investigations of the SGCS powders were performed for analyzing the chemical composition and binding states of the elements on the outermost surfaces of the catalysts. Table S1 shows the results of the XPS survey spectra calculated as the average elemental content on the surface of the catalysts. According to the data, the outermost surfaces of all the synthesized catalysts contained contaminants such as C, O, and N. The C, O, and N contents were in the 47–59 at. % range, 19–32 at. % range, and 7–15 at. % range, respectively. These elements were the decomposition products of the initial precursors and could be readily removed by short-term heat treatment [46].

More importantly, the Co/Ni ratio in the near-surface layers (5–10 nm, analyzed by XPS) of the bimetallic catalysts appeared to be higher, compared with the stoichiometric composition (i.e., 5.2 vs. 3.0; 1.9 vs. 1.0; and 0.8 vs. 0.33, for Co_3_Ni, CoNi, and CoNi_3_ alloys, respectively). However, the ratios measured by EDS diagnostics (average from the volume on a scale of ~2 µm) agreed well with expected stoichiometry results (Figure S1).

**Figure 3:**
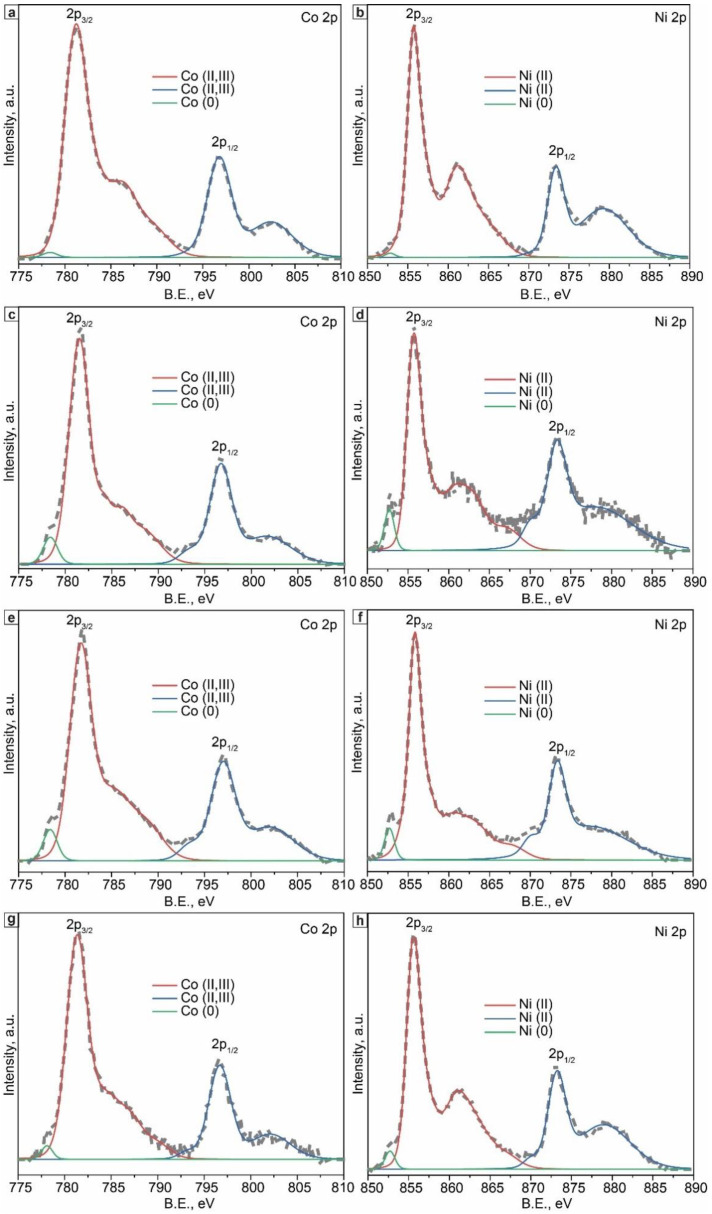
High-resolution XPS spectra of the as-synthesized (**a**) Co, (**b**) Ni, (**c**,**d**) Co_3_Ni, (**e**,**f**) CoNi, and (**g**,**h**) CoNi_3_ catalysts.

The Co and Ni binding states within the near-surface region of the as-synthesized catalysts were studied by analyzing high-resolution XPS spectra of the Co 2p and Ni 2p energy levels (Figure 3). The Co 2p and Ni 2p spectra of all the samples were fitted into two main binding-energy signals of Co 2_3/2_, Co 2p_1/2_, and Ni 2p_3/2_, Ni 2p_1/2_, respectively. The former two signals were assigned to the core levels of Co(III) and Co(II), while the latter was associated with the Ni(II) oxidative state. Low-intensity metallic peaks in the Co(0) and Ni(0) states were also observed for all the investigated catalysts. Recall that the XRD analysis revealed only metallic phases.

### 3.2. In Situ DRIFTS Experiments

Elicitation of the active sites for the analyzed single-phase and bimetallic catalysts was performed using DRIFTS-CO/CO_2_ analysis. The similar behavior of all bands of the C-O stretching vibrations in the spectra of single Co and Ni catalysts did not allow the proper assignment of the bands in the spectra of the bimetallic samples (Figure S2). However, analysis of the DRIFT-CO spectra for single Co and Ni catalysts clearly elucidated (Figure S2) the presence of 2006, 2059, 2104, 2128, 2177 cm^−1^, and 2010, 2056, 2114, 2166, 2222 cm^−1^ bands, respectively. The presence of those bands identified few different Co and Ni electronic states, such as Co^2+^-CO (2177 and 2128 cm^−1^), Co^+^-CO (2128 cm^−1^), Co^δ+^-CO (2104 cm^−1^), Co^0^-CO (2059 and 2006 cm^−1^) [2,3,4,5,6,7,8,9,10,11,12,13,14,15,16] and Ni^2+^-CO (2222 and 2166 cm^−1^), Ni^+^-CO (2114 cm^−1^), Ni^δ+^-CO (2056 cm^−1^), and Ni^0^-CO (2010 cm^−1^) [2,3,17,18,19,20,21,22,23,24,25]. All of the data agreed well with the XPS results. The CO adsorption was fully reversible for the single-metal catalysts, and only a small amount of CO remained on the alloy catalyst surfaces after the finalizing vacuum treatment at room temperature.

**Figure 4:**
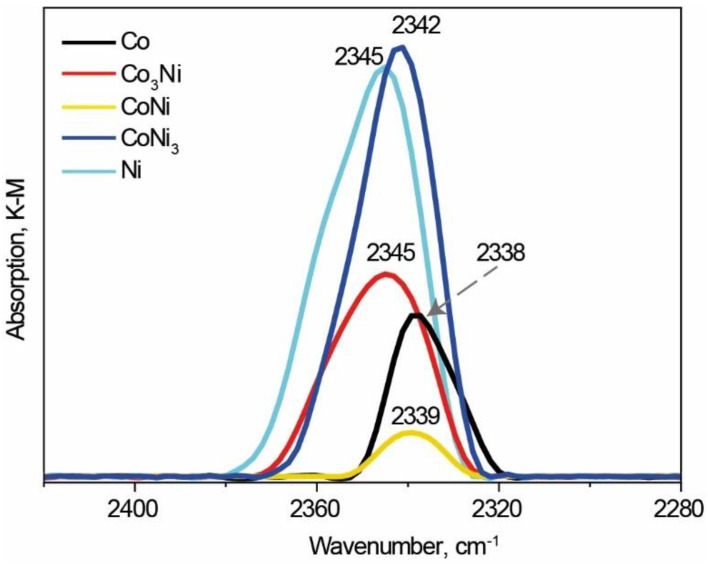
DRIFT-CO_2_ spectra of Co, Ni, and bimetallic Co_x_Ni_1−x_ catalysts.

In the case of the CO_2_ adsorption on the catalysts with different Co and Ni compositions, a few weak spectra were observed in the 2400–2300 cm^−1^ range of wavelengths (Figure 4). The corresponding bands were at 2338 cm^−1^, 2345 cm^−1^, 2339 cm^−1^, 2345 cm^−1^, and 2342 cm^−1^ for the Co, Ni, Co_3_Ni, CoNi, and CoNi_3_ samples, respectively. These bands corresponded to the asymmetric valence υ_3_ vibrations of the adsorbed CO_2_ molecules. The shift direction of these bands can be explained by the strong polarization of CO_2_ molecules adsorbed on the alloy surfaces, which interacted with the cations [53].

In this study, the 2338 cm^−1^ band was used as a standard; thus, a positive shift from 1 to 7 cm^−1^ was observed for every sample except for the monometallic Co catalyst. These results indicate stronger adsorption of CO_2_ molecules on the Ni, Co_3_Ni, and CoNi_3_ surfaces. The higher intensity of the lines in the Ni and CoNi_3_ spectra indicated a larger number of adsorbed CO_2_ molecules on the surfaces of these samples. After the vacuum treatment at room temperature, the CO_2_ spectra disappeared for the single-metal samples and are became less intense without any shift for the bimetallic CoNi and CoNi_3_ samples, while a slight shift was observed for Co_3_Ni (Figure S3).

### 3.3. Temperature-Programmed Reduction Experiments

**Figure 5:**
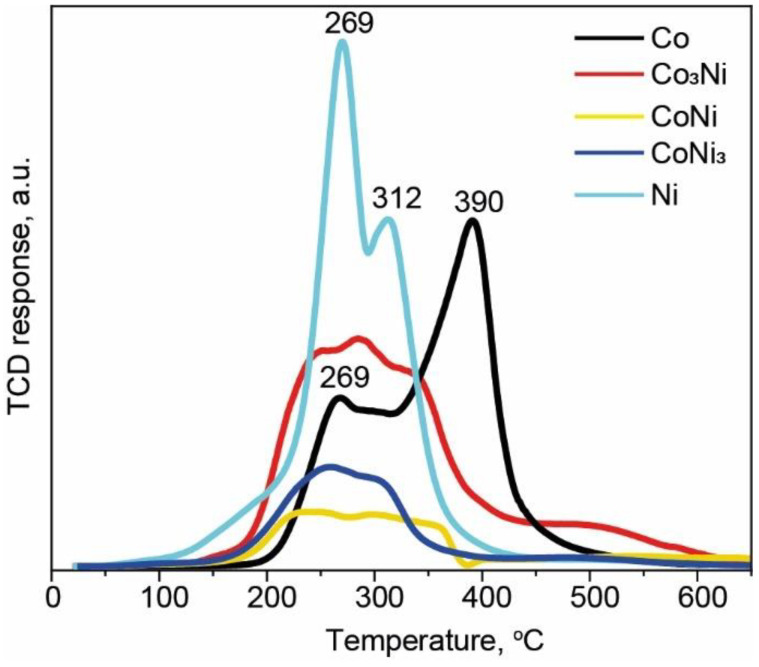
H_2_-TPR profiles of Co, Ni, and bimetallic Co_x_Ni_1−x_ catalysts.

H_2_-TPR studies were performed to elucidate the surface reducibility of the prepared Co, Co_3_Ni, CoNi, CoNi_3_, and Ni catalysts (Figure 5). All TPR profiles are characterized by a wide range of H_2_ consumption temperatures, which indicates the occurrence of several parallel reduction processes on the catalytic surface. The Co reduction profile exhibited two characteristic peaks at 269 °C and 390 °C. These results corresponded to the consistent reduction in Co oxides from Co^3+^ to Co^2+^ and from Co^2+^ to Co^0^ [54]. There were three peaks in the reduction profile of the Ni catalyst, with two of them in the 100–400 °C range, indicating desorption of C- and N-containing residuals, as well as chemisorbed water, which were detected by the XPS analysis. The third peak at 312 °C could be attributed to the reduction of Ni^2+^ to Ni [55].

All the bimetallic catalysts demonstrated lower H_2_ consumption intensities, compared with the Co and Ni catalysts. These results suggest that the surfaces of the Co_x_Ni_1−x_ alloys are less contaminated and more stable with respect to oxidation.

### 3.4. CO_2_ Hydrogenation Performance

Catalytic activities of the as-synthesized Co, Co_3_Ni, CoNi, CoNi_3_, and Ni catalysts were evaluated for CO_2_ hydrogenation at 300 °C. The experimental conditions at such temperature ensured the maximal thermodynamic conversion of CO_2_ into CH_4_. All the samples demonstrated exceptional selectivity with respect to the CH_4_ formation, which was close to 100% (Table 2). Water was the main byproduct of the reaction, and only trace amounts of CO were detected.

The Co catalyst exhibited a slightly higher efficiency with respect to the CH_4_ formation calculated per gram (0.12 mol·h^−1^·g_cat_^−1^) of the active component, compared with the Ni catalyst (0.09 mol·h^−1^·g_cat_^−1^). Nevertheless, bimetallic Co_x_Ni_1−x_ alloy-based catalysts exhibited twofold to threefold higher activity, where the CoNi_3_ was the most active composition (0.27 mol·h^−1^·g_cat_^−1^). The values for the CH_4_ efficiency formation over the bimetallic catalysts were similar, but they tended to increase with increasing the Ni content (Table 2). The CO_2_ conversion observed for the alloy catalysts was approximately 89%, much higher than for pure metals, that is, 67% and 72% for Ni and Co, respectively.

### 3.5. Catalyst Stability and Productivity Test

**Figure 6:**
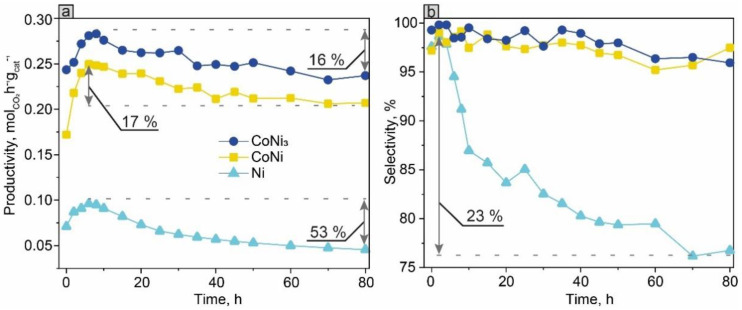
Productivity (**a**) and CH_4_ selectivity (**b**) for the CoNi, CoNi_3_, and Ni catalysts, during the 80-h-long CO_2_ hydrogenation reaction at 300 °C.

CoNi_3_ and CoNi alloys with the highest catalytic activity were further used for assessing the stability of bimetallic bulk catalysts compared with the Ni catalyst, which is considered to be one of the basic components for producing synthetic natural gas by the hydrogenation of CO_2_. Figure 6 shows the activity and selectivity of the CO_2_ hydrogenation reaction with respect to CH_4_, over the 80-h-long TOS at 300 °C. The samples reached maximal catalytic activity after 6 h from the beginning of the experiment (Figure 6a). The Ni catalyst began to lose its activity monotonously throughout the reaction. The maximal decrease was 53% at 80 h of TOS. Simultaneously, the selectivity of CH_4_ dropped to 76%, and the CO fraction became more visible.

Evidently, alloy-based catalysts exhibited much better stability. After 80 h of TOS, the catalytic activity dropped only by ~16% both for the CoNi and CoNi_3_ alloys. It is worth noting that a slight decrease in activity (by 15–20%) during the first hours of the run is expected for activated catalysts [56]. At the same time, the extremely high selectivity (>99%) with respect to the CH_4_ formation remained constant throughout the experiment, indicating the stability of the catalytic properties of the active sites.

## 4. Discussion

### 4.1. CO_2_ Hydrogenation Performance

All of the above-discussed bimetallic bulk catalysts exhibited high catalytic activity with respect to the CO_2_ hydrogenation reaction, comparable to the most effective reported catalysts [29]. However, most of the reported results are related to supported catalysts. In addition, for supported monometallic catalysts, Ni is typically more active than Co [57]. In our case, the bulk monometallic Co catalyst prepared by combustion was more active than the Ni-based catalyst. This conclusion is in agreement with the DRIFT data (Figure 4), where a stronger adsorption of CO_2_ was observed on the Ni surface than on the Co bulk catalyst. Moreover, the porosity of the Co catalyst was significantly higher than that of Ni (Figure 2), which might have reduced the diffusion constraint. Such a result shows that the dependence of the catalytic activity on the nature of the active metal is not straightforward, as is commonly believed. The mechanism of the CO_2_ transformation depends on the supporting material [57,58,59] and can be affected by the morphology of the catalytic constituents.

A more important observation was that a combination of Co and Ni constituents led to a synergistic effect with respect to the catalytic activity of bulk alloy catalysts. Bulk alloy catalysts exhibited twofold to threefold higher activities than monometallic SGCS catalysts (Table 2). The crystallite size of the SGCS catalysts was approximately 30–40 nm (Table 1), higher than that of the supported analogues (5–10 nm) [60]. Hence, based on the commonly used paradigm, the catalytic activity of bulk Co-Ni materials should be much lower than that of supported materials. Nevertheless, the activity of the SGCS Co_x_Ni_1−x_ catalysts was comparable to or even higher than that of many reported supported catalysts. The active phase content of supported Ni/Co-based catalysts usually does not exceed 20 wt % [29,61], which is close to that used in this work. However, in the case of the SGCS sponge-like catalysts, the catalytically active surface was potentially equivalent to the overall surface of the 100 µm diameter metallic sponge-like particles.

In addition, comparing the results of the long-term tests, it can be concluded that the introduction of Co stabilized the catalyst properties, protecting it from deactivation (Figure 6). The XPS results showed that before the catalysis, the Co concentration on the outermost surface of the SGCS alloy catalysts was higher than that of Ni, even when the stoichiometric ratio of the constituents was kept in a larger volume. A similar trend has been observed in other studies [42]. However, after the catalysis, the amount of Ni on the surface increased (Table S1). Thus, it may be suggested that owing to the higher affinity for O, Co protected the Ni active sites from oxidation during the reaction (see also [48]). The H_2_-TPR results support this conclusion, showing the better oxidation resistance of the alloys relative to pure Ni (Figure 5).

In addition, the majority of publications on CO_2_ hydrogenation outline the problem of the low thermal stability of the catalysts and their fast deactivation [59]. The application of catalytically active elements on the surface of an inert supporting material is usually considered a universal solution of this problem. This tendency has historical roots when the approach is used to optimize catalysts based on noble metals [62]. Still, in the case of the CO_2_ hydrogenation on the Ni-supported catalyst, significant difference between the thermal conductivities of Ni (90.7 W/m·K) and ceramic supports (e.g., CeO_2_ at 8 W/m·K [28]) could yield local hot spots and subsequent carbonization of active sites [11,29]. In this work, active sites were distributed on relatively large (100–500 µm) metallic particles, which provided additional space and time for extensive heat dissipation. Moreover, the unique morphology of these particles generated during combustion synthesis manifested as a highly porous sponge structure and an additional instrument for fast heat and mass transfer. These features positively affected the stability of the SGCS catalysts.

Thus, it can be concluded that the catalytic efficiency of one active metal can be significantly enhanced by the addition of another active metal. More importantly, alloys show much higher stability than pure catalysts. Indeed, the bulk Ni catalyst prepared using an energy-saving combustion approach was stabilized by the addition of another catalytically active metal such as Co. In addition, it is important to note that typical oxide-based supports stabilize the metal oxide phase, leading to a high reduction temperature [63]. For example, industrial activation of Ni-based catalysts occurs at 400 °C, while the reduction process of the catalysts synthesized in this work already ended at 350 °C (Figure 5). Thus, the implementation of the SGCS bulk alloy catalysts might be a step toward the simplification of the catalyst production approach and the higher cost efficiency of the CO_2_ hydrogenation reaction. However, to control the process, it is critical to understand the hydrogenation mechanism associated with the catalysts used.

### 4.2. Comments on the Possible CO_2_ Hydrogenation Mechanism

The CoNi_3_ catalyst was chosen as the most active catalyst for investigating the CO_2_ hydrogenation reaction mechanism. The presence of CH_4_ and CO as the main reaction products suggests two viable reaction paths: the formation of CH_4_ through the CO intermediate (overall sequence of Reactions (6) and (7)) or directly from CO_2_ (overall Reaction (8)):

CO_2_ + H_2_ → CO + H_2_O (6)

CO + 3H_2_ → CH_4_ + H_2_O (7)

CO_2_ + 4H_2_ → CH_4_ + 2H_2_O (8)

**Figure 7:**
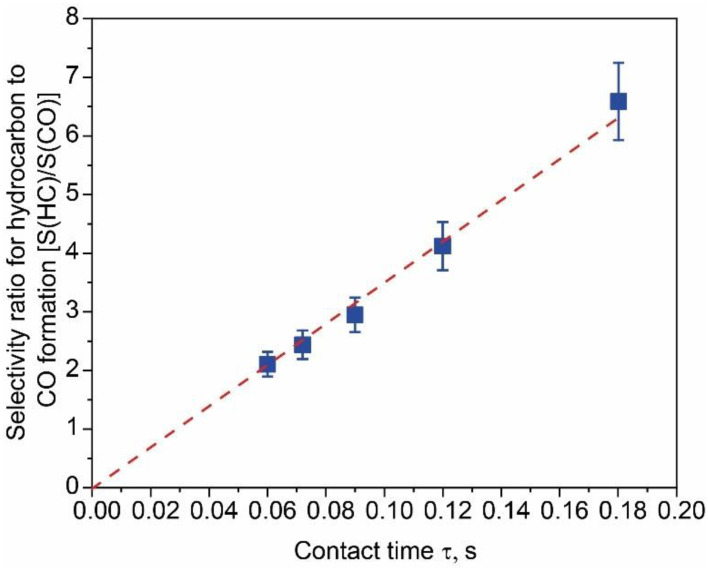
The ratio between HC and CO selectivity as a function of the contact time.

Figure 7 shows the selectivity ratio of hydrocarbons to CO formation as a function of the contact time of the feeding mixture with the CoNi_3_ catalyst. It can be clearly observed that at a lower contact time, the S(HC)/S(CO) ratio tends to zero. This observation suggests that in our case, CO formed first (Reaction (6)) and later transformed to CH_4_ (Reaction (7)).

**Figure 8:**
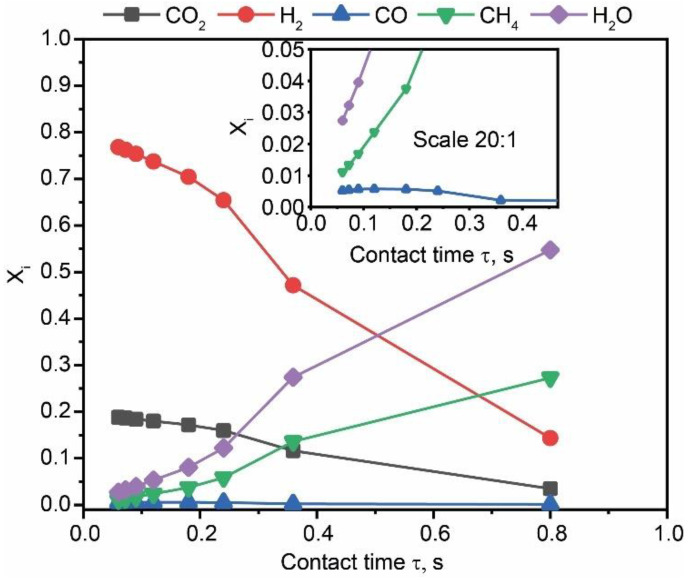
Dependence of the concentration of precursors and products as a function of the contact time.

The dependence of the concentration of gaseous precursors and products on contact time is shown in Figure 8. The concentration of the feeding gas reagents decreases with time, while the concentrations of CH_4_ and H_2_O products constantly increase. The value of the CO gas concentration reaches a maximum and then decreases toward zero. This behavior is characteristic of the final product formation through consecutive reactions. Thus, we hypothesize that the formation of CH_4_ from CO_2_ involves an intermediate stage of the CO formation. It should be noted that even at its peak, the CO concentration was twice lower that of the CH_4_ concentration. This means that the reaction rate of the CO formation was significantly lower than that of the CH_4_ formation. Thus, the CO formation reaction is the limiting stage of the overall process.

Finally, it has been reported that the distinctive feature of Co, compared with Ni, is its ability to catalyze not only direct CO_2_ methanation but also the reverse water shift reaction, with the formation of CO [58,64]. Therefore, it is possible that the presence of Co enhances the rate of the CO gas formation, which is later converted to CH_4_. Moreover, according to the DRIFT-CO_2_ analysis, the presence of Co decreases CO_2_ adsorption on the surface of the alloy and thus protects the Ni active sites from poisoning through the formation of solid C layers, which blocks the access of the feeding gas to the Ni active sites.

## 5. Conclusions

In this study, bulk single-phase Co-Ni alloy catalysts with broad Co_x_Ni_1−x_ composition (x = 1, 0.75, 0.5, 0.25, and 0) were synthesized by the combustion of reactive sol-gels. The catalysts had a sponge-like microstructure and a highly crystallized intermetallic FCC crystal structure. The overall desired stoichiometric Co/Ni ratio was well-preserved in the volume of the materials, while the near surface of the Co_x_Ni_1−x_ alloys saturated twice with Co. Gradual addition of Co into Ni allowed the governing of the alloy microstructure and obtaining a more porous structure and a smaller intermetallic crystallite size.

The combination of Co and Ni in a single phase resulted in a synergistic effect with respect to the catalytic activity, showing threefold higher efficiency than that of single-metal catalysts. The CoNi_3_ composition was the most active catalyst with efficiency to the CH_4_ formation of 0.27 mol·h^−1^·g_cat_^−1^ at VHSV = 4500 mL·g^−1^h^−1^. It was also demonstrated that the Co constituent stabilized the catalyst toward deactivation. At the same time, the selectivity for 99+% methane formation on the alloy samples remained constant throughout the experiment. Moreover, the bimetallic catalysts demonstrated lower H_2_ consumption intensity than the monometallic catalysts, indicating that the alloys are more stable to oxidation.

A study of the relationship between the concentration of both the feeding mixture and gaseous products on the catalyst surfaces during the hydrogenation process allows us to suggest the CO_2_ hydrogenation mechanism. This implies that the overall hydrogenation proceeds in a sequence of steps. First, the Co active sites facilitate the formation of gaseous CO, followed by its conversion to CH_4_. Moreover, the presence of Co decreases the CO_2_ adsorption on the Co_x_N_1−x_ surface and hence protects the Ni active sites from oxidation and formation of solid C particles, i.e., poisoning of the catalyst.

The fact that the activity of SGCS Co_x_Ni_1−x_ catalysts is comparable or even higher than that of supported catalysts suggests that the implementation of bulk sponge-like porous alloys can be a step toward simplification of catalyst production technology and the development of an efficient approach to handle the problem of CO_2_ emission into the atmosphere.

## Figures and Tables

**Table 1:** Structure characteristics of the catalysts.

Sample ID	Crystallite Size (D), nm	Lettice Constant (a), Å
Co	29	3.5457
Co_3_Ni	27	3.5349
CoNi	32	3.5336
CoNi_3_	37	3.5246
Ni	38	3.5233

**Table 2:** Catalytic performance of the CO_2_ hydrogenation reaction, for the Co, Co_3_Ni, CoNi, CoNi_3_, and Ni catalysts (calculated at T = 300 °C, P = 2 MPa, H_2_:CO_2_ = 4:1, VHSV = 4500 mL·g^−1^h^−1^).

Sample ID	CH_4_ Selectivity, %	Efficiency by CH_4_, mol·h^−1^·g_cat_^−1^	CO_2_ Conversion, %
Co	99+	0.12	71.5
Co_3_Ni	99+	0.21	89.1
CoNi	99+	0.24	88.4
CoNi_3_	99+	0.27	88.7
Ni	99+	0.09	66.6

## Supplementary Materials

The following supporting information can be downloaded at https://www.mdpi.com/article/10.3390/ma15155129/s1: Figure S1: EDS spectra of Co_3_Ni (a), CoNi (b), and CoNi_3_ (c) catalysts; Table S1: XPS average elements content in the outermost surface of the Co-Ni catalysts; Figure S2: DRIFT-CO spectra of single Co, Ni, and bimetallic Co_x_Ni_1−x_ catalysts; Figure S3: DRIFT-CO_2_ absorption-desorption spectra of Co (a), Ni (b), Co_3_Ni (c), CoNi (d), and CoNi_3_ (e).
