# Correction: Evdokimenko et al. Sponge-like CoNi Catalysts Synthesized by Combustion of Reactive Solutions: Stability and Performance for CO_2_ Hydrogenation. *Materials* 2022, *15*, 5129

**DOI:** 10.3390/ma18225175

**Published:** 2025-11-14

**Authors:** Nikolay Evdokimenko, Zhanna Yermekova, Sergey Roslyakov, Olga Tkachenko, Gennady Kapustin, Denis Bindiug, Alexander Kustov, Alexander S. Mukasyan

**Affiliations:** 1 Center of Functional Nano-Ceramics, National University of Science and Technology “MISiS”, 119049 Moscow, Russia; 2 N.D. Zelinsky Institute of Organic Chemistry RAS, 119991 Moscow, Russia; 3 Department of Chemistry, M. V. Lomonosov Moscow State University, 119991 Moscow, Russia; 4 Department of Chemical and Biomolecular Engineering, University of Notre Dame, Notre Dame, IN 46556, USA

In the original publication [1], there was an overlap in Figure 2 as published. The corrected Figure 2 appears below. The authors state that the scientific conclusions are unaffected. This correction was approved by the Academic Editor. The original publication has also been updated.

## Figures and Tables

**Figure 2:**
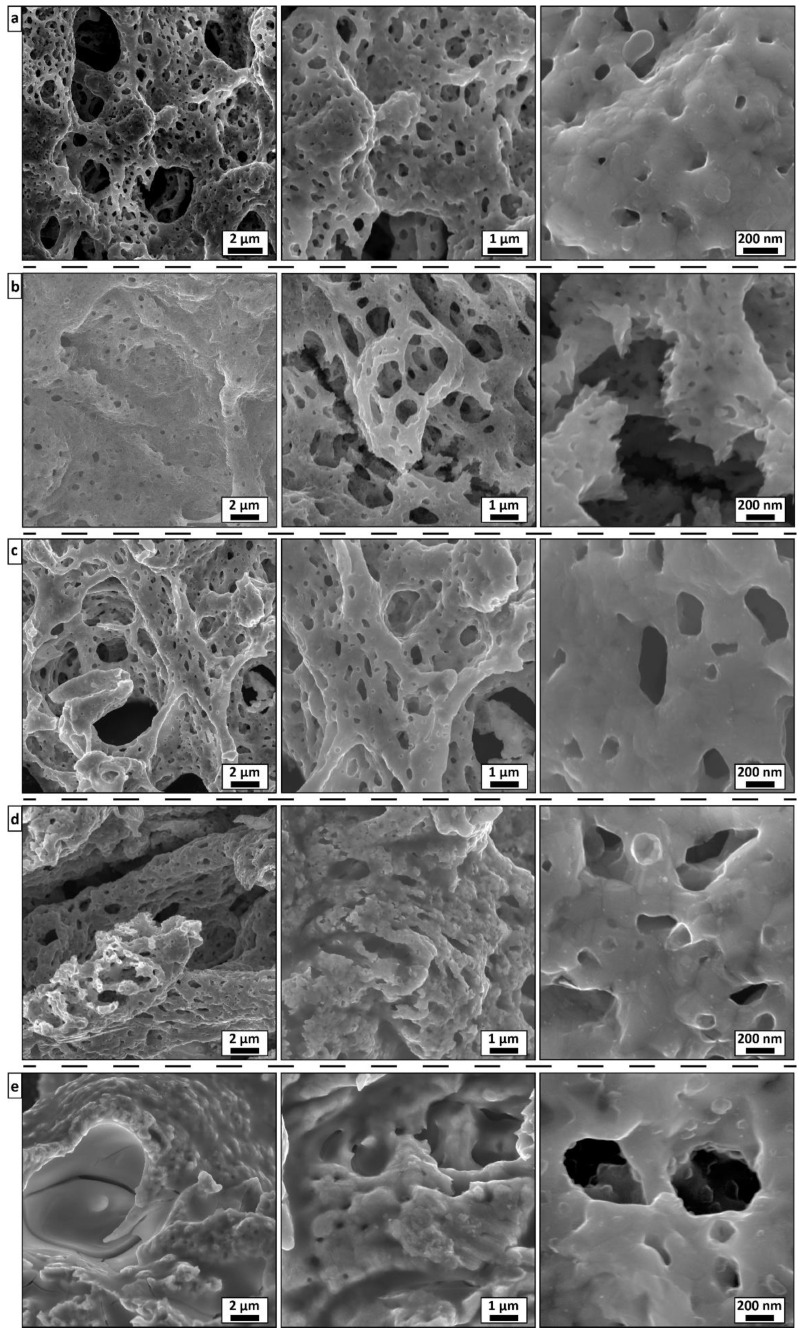
SEM images of (**a**) Co, (**b**) Co_3_Ni, (**c**) CoNi, (**d**) CoNi_3_, and (**e**) Ni bulk catalyst powders synthesized by SGCS.
