# Supplementary material for: Sponge-like CoNi Catalysts Synthesized by Combustion of Reactive Solutions: Stability and Performance for CO2 Hydrogenation
Source: Materials (Basel). 2022 Jul 23;15(15):5129. doi: 10.3390/ma15155129 (PMC9329901; doi:10.3390/ma15155129)
Supplement: Supplementary file 1 [file materials-15-05129-s001.zip › materials-1802419-supplementary.pdf]

materials

Supplementary Material

Sponge-like CoNi Catalysts Synthesized by Combustion of Reactive Solutions: Stability and Performance for CO_2 Hydrogenation

Nikolay Evdokimenko [1,2], Zhanna Yermekova [1], Sergey Roslyakov [1,*], Olga Tkachenko [2], Gennady Kapustin [2], Denis Bindiug [1], Alexander Kustov [1,2,3] and Alexander S. Mukasyan [4,*]

1 Center of Functional Nano-Ceramics, National University of Science and Technology "MISiS", 119049 Moscow, Russia; nikolayevdokimenko@bk.ru (N.E.); ifjanna@gmail.com (Z.Y.); denis-bindyug@yandex.ru (D.B.); kyst@list.ru (A.K.)
2 N.D. Zelinsky Institute of Organic Chemistry RAS, 119991 Moscow, Russia; ot113@mail.ru (O.T.); gik@server.ioc.ac.ru (G.K.)
3 Department of Chemistry, M. V. Lomonosov Moscow State University, 119991 Moscow, Russia
4 Department of Chemical and Biomolecular Engineering, University of Notre Dame, Notre Dame, IN 46556, USA
* Correspondence: roslyakov.si@misis.ru (S.R.); amoukasi@nd.edu (A.S.M.)

Table S1. XPS average elements content in the outermost surface of the Co-Ni catalysts.

Catalysts	Element content, at. %					Co/Ni atomic ratio
	O	C	N	Co	Ni	
Co, as-synthesized	26.0	58.5	7.2	8.3	–	–
Co_3Ni, as-synthesized	23.4	47.3	15.5	11.6	2.2	5.2
CoNi, as-synthesized	19.0	56.6	13.5	7.2	3.7	1.9
$CoNi_3$, as-synthesized	28.5	48.9	10.3	5.5	6.8	0.8
Ni, as-synthesized	32.5	52.6	8.4	–	6.5	–
$CoNi_3$, spent	35.0	38.6	–	11.1	15.3	0.7
CoNi, spent	48.4	26.3	–	15.2	10.1	1.5
Ni, spent	30.0	56.1	–	–	13.9	–

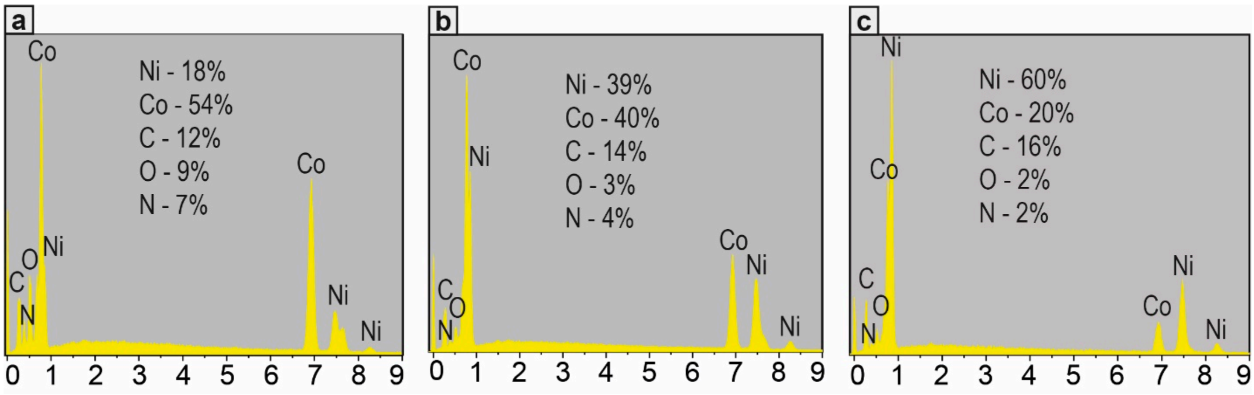

Figure S1. EDS spectra of Co_3Ni (a), CoNi (b), and $CoNi_3$ (c) catalysts.

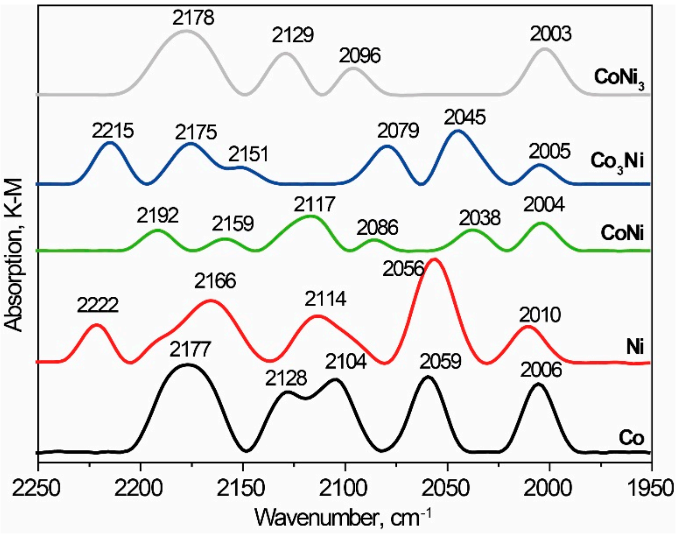

Figure S2. DRIFT-CO spectra of single Co, Ni, and bimetallic Co_xNi_{1-x} catalysts.

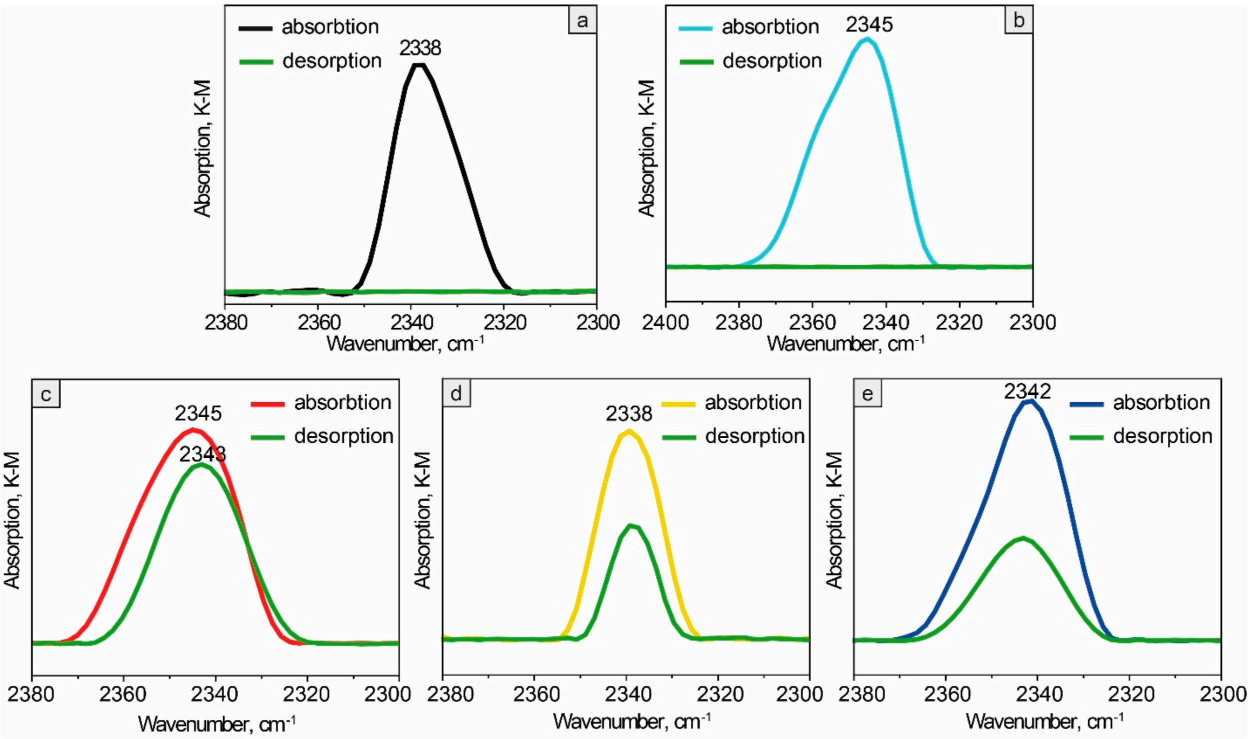

Figure S3. DRIFT-CO_2 absorption-desorption spectra of Co (a), Ni (b), Co_3Ni (c), CoNi (d), and $CoNi_3$ (e).
